# Rosendaal linear interpolation method appraising of time in therapeutic range in patients with 12-week follow-up interval after mechanical heart valve replacement

**DOI:** 10.3389/fcvm.2022.925571

**Published:** 2022-09-09

**Authors:** Xiliang Zhu, Xijun Xiao, Sheng Wang, Xianjie Chen, Guoqing Lu, Xiaoyang Li

**Affiliations:** ^1^Department of Cardiovascular Surgery, Henan Province People’s Hospital, Zhengzhou University, Zhengzhou, Henan, China; ^2^Department of Cardiovascular Surgery, West China Hospital, Sichuan University, Chengdu, China

**Keywords:** warfarin, time in therapeutic range (TTR), international normalized ratio (INR), mechanical heart valve (MHV), oral anticoagulant

## Abstract

**Background:**

The objective of this study was to evaluate the quality of anticoagulation by the time in therapeutic range (TTR) for patients with 12-week INR follow-up interval.

**Materials and methods:**

From January 2018 to December 2020, a selective group of patients who underwent mechanical valve replacement and followed up at our anticoagulation clinic for adjustment of warfarin dose were enrolled. The incidences of complications of anticoagulation therapy were reported by linearized rates. TTR was calculated by the Rosendaal linear interpolation method.

**Results:**

Two hundred and seventy-four patients were eligible for this study. The mean age of these patients was 52.8 ± 12.7 years, and 65.7% (180 cases) of them were females. The mean duration of warfarin therapy was 16.7 ± 28.1 months. A total of 1309 INR values were collected, representing 66789 patient days. In this study, the mean TTR was 63.7% ± 18.6%, weekly doses of warfarin were 20.6 ± 6.0 mg/weekly, and the mean monitoring interval for the patient was 53.6 ± 27.1 days. There were 153 cases in good TTR group (TTR ≥ 60%) and 121 cases in poor TTR group (TTR < 60%). The calculated mean TTR in both groups was 42.6% ± 22.1% and 74.8% ± 10.4%, respectively. Compared with the TTR ≥ 60% group, the TTR < 60% group exhibited a more prevalence of female gender (*p* = 0.001), atrial fibrillation (*p* < 0.001), NYHA ≥ III (*p* < 0.001), and lower preoperative left ventricular ejection fraction (LVEF, *p* = *0.032*). In multivariate analysis, female gender (*p* = 0.023) and atrial fibrillation (*p* = 0.011) were associated with TTR < 60%. The incidence of major bleeding and thromboembolic events was 2.7% and 1.1% patient-years, respectively. There was one death which resulted from cerebral hemorrhage. The incidence of death was 0.5% patient-years. The difference in anticoagulation-related complications between the TTR < 60% group and the TTR ≥ 60% group was not statistically significant.

**Conclusion:**

For patients with stable international normalized ratio monitoring results who are follow-up at anticoagulation clinics, a 12-week monitoring interval has an acceptable quality of anticoagulation. The female gender and atrial fibrillation were associated with TTR < 60%.

## Introduction

Patients with mechanical heart valve prostheses need to receive oral anticoagulant therapy for life long, or they may accompany with valve thrombosis and subsequent systemic embolism (1). Warfarin, a common vitamin K antagonist, has been demonstrated to be effective for the prevention and treatment of those thromboembolic complications (2, 3). However, its limitations are also remarkable, including a narrow therapeutic range, intra- and inter-patient variability in dose response, and susceptibility to drug–drug and drug–food interactions (1, 4). For this reason, it is important to periodically monitor and adjust the dose to keep the international normalized ratio (INR) in the target range as long as possible.

The interval between INR monitoring and stable doses of oral anticoagulants continues to be controversial (5). The American College of Chest Physicians (ACCP) recommends 4 weeks as the maximum interval for patients’ follow-up. Other studies suggest that it can be extended to 12 weeks for patients with stable INR monitoring results and point out that every 4-week follow-up may increase the possibility of testing results out of the target range and the burden of anticoagulant treatment in these patients (6–9). The clinical outcomes of 12-week intervals of assessment have been found to be safe and to be equal to assessments every 4 weeks in other studies (7, 9). Despite this, there is still a lack of evidence supporting the longer monitoring interval in the literature or in clinical practice.

Time in the therapeutic range (TTR), which estimates the percentage of time a patient’s INR is within the therapeutic treatment range or goal, is commonly used in the assessment of anticoagulant quality (8, 10–14). Studies indicated that patient with higher TTR is directly correlated with the reduction in risk of thromboembolism complications and major bleeding (15, 16). Lee et al. (14) showed that a 10% increase in time spent out of TTR is related to a 10–12% decrease in the risk thromboembolic events and a 29% decrease in the risk of mortality. Therefore, the objective of this study was to evaluate the quality of anticoagulation in our hospital by the TTR for patients with 12-week INR monitoring interval and to identify the predictors of poor TTR in patients with mechanical heart valve prostheses.

## Materials and methods

### Patients

The retrospective research was conducted in Henan Province People’s Hospital. From January 2018 to December 2020, a selective group of patients who received warfarin treatment and followed up at our anticoagulation clinic were enrolled. All of them had undergone mechanical valve replacement. Patients were seen as qualified to participate in the research if they meet the following criteria: (1) aged at 18 years old or above, (2) accepted warfarin treatment for at least 12 months, and (3) had a stable dose for the previous 12 weeks. Patients were excluded from this study under the following conditions: (1) monitoring interval > 13 weeks; (2) ethnic minorities of China.

Referring to our anticoagulation clinic’s anticoagulant criteria, the target range for INR in our hospital is 1.5 to 2.5. INR < 1.5 is defined as subtherapeutic range while INR > 2.5 as supratherapeutic range. We adjust the dose and monitoring intervals according to the protocol in Table 1. We recommend 4 weeks as the maximum monitoring interval for patients who underwent mechanical heart valve replacement within postoperative 6 months. After 6-month intensive INR monitoring, 12 weeks is recommended to the patient who keep a stable dose for 12 weeks.

**TABLE 1:** Dose adjustment protocol according to international normalized ratio (INR) value in our anticoagulation clinical.

INR value	Action
<1.5	Increasing the dosage of 0.625 mg/day
1.5–2.5	Maintain dose
>2.5 and ≤3.5	Omit one dose, and reducing the dosage of 0.625 mg/day, weekly control until level stabilizes
>3.5	Omit three dose, and reducing the dosage of 0.625 mg/day, weekly control until level stabilizes

Patient with INR >5 is referred to the emergency department. INR: international normalized ratio.

### Time in therapeutic range

Time in therapeutic range (TTR) is defined as the number of person-day within a pre-determined therapeutic range divided by the total number of person-days on warfarin (10, 17). TTR was calculated by the Rosendaal linear interpolation method that assumes a linear relationship exists between two INR values and allows one to allocate a specific INR value to each day for each patient (10, 17). Enrolled patients were divided into the “poor” TTR (TTR < 60%) group and the “good” TTR (TTR ≥ 60%) group. The clinical characteristics of patients are shown in Table 2.

**TABLE 2:** Clinical characteristics of patients.

Characteristics	Value
Age, years	52.8 ± 12.7
Female, n (%)	180 (65.7%)
Diabetes mellitus, n (%)	32 (11.7%)
Hypertension, n (%)	41 (15.0%)
Smoking, n (%)	48 (16.8%)
NYHA ≥ III, n (%)	205 (74.8%)
Creatinine, μmoI/L	73.4 ± 17.8
Total cholesterol, mmol/L	3.2 ± 0.7
HbA1c, %	6.1 ± 1.0
Echocardiography	
LVEDD, mm	48.1 ± 4.7
LVEF, %	59.5 ± 5.9
Operations	
MVR, n (%)	137 (50.0%)
MVR and AVR, n (%)	94 (34.3%)
AVR, n (%)	43 (15.7%)
Number of INR values	4.8 ± 1.6
weekly doses of warfarin	20.6 ± 6.0

AVR, aortic valve replacement; MVR, mitral valve replacement; LVEDD, left ventricular end-diastolic dimension; LVEF, left ventricular ejection fraction; NYHA, New York heart association.

### Data collection

The approach to follow-up is to review the electronic record system and conduct a telephone interview. The clinical data of patients include the type of mechanical valve, age, gender, INR values, intervals of INR monitoring, duration of anticoagulant management, warfarin dose, and anticoagulation-related complications.

Anticoagulation-related complications included major bleeding events, thromboembolic events, and death. Major bleeding events were defined as bleeding that should receive hospital treatment, such as cerebral hemorrhage, gastrointestinal hemorrhage, and hematuria. Other bleeding events, including epistaxis, gingival bleeding, and ecchymosis, were classified as minor bleeding events. Thromboembolic events include cerebral vascular embolism, systemic embolism, and atrial thrombosis. Death was evaluated by the medical record or a death certificate for relationship with bleeding or thromboembolism.

This retrospective study followed the tenets of Declaration of Helsinki and was approved by the ethics review board of Henan Province People’s Hospital. A waiver of consent was received from them at the same time.

### Statistical analysis

Continuous variables were described in the form of means ± standard deviation (SD) and were analyzed with Student’s *t*-test. Categorical data were reported as rates and were analyzed with the chi-squared test. Independent predictors for TTR < 60% were evaluated with the logistic regression model. Variables with *p* < 0.20 in univariate analysis were incorporated into multivariate analysis. A two-sided *p* < 0.05 was regarded as statistically significant. The incidences of valve-related complications were reported by linearized rates. All data were analyzed with Statistical Package for Social Sciences (SPSS V17.0, Chicago, Illinois, United States).

## Results

Two hundred and seventy-four patients were eligible for this study. As shown in Table 2, patients are classified based on their clinical characteristics. The mean age of these patients was 52.8 ± 12.7 years, 65.7% (180 cases) of them were female and 34.3% (94 cases) were male. The mean duration of warfarin therapy was 16.7 ± 28.1 months. Operations of these patients were as follows: 50.0% (137 cases) patients were performed mitral valve replacement (MVR), 15.7% (43 cases) were performed aortic valve replacement (AVR), and 34.3% (94 cases) were performed MVR and AVR.

A total of 1309 INR values were collected, representing 66789 patient days, each patient with a mean of 4.8 ± 1.6 INR values. In this study, the mean TTR was 63.7% ± 18.6%, weekly doses of warfarin were 20.6 ± 6.0 mg/weekly, and the mean monitoring interval for the patient was 53.6 ± 27.1 days. There were 153 cases in good TTR group (TTR ≥ 60%) and 121 cases in poor TTR group (TTR < 60%). The calculated mean TTRs for both group were 42.6% ± 22.1% and 74.8% ± 10.4%.

Univariate analyses of preoperative variables associated with TTR < 60% are shown in Table 3. Compared with the TTR ≥ 60% group, the TTR < 60% group exhibited a more prevalence of female gender (*p* = 0.001), atrial fibrillation (*p* < 0.001), NYHA ≥ III (*p* < 0.001), and lower preoperative left ventricular ejection fraction (LVEF, *p* = 0.032). The mean age in the TTR ≥ 60% group was 51.9 ± 13.8, and the mean age in the TTR < 60% group was 53.3 ± 11.2; there was no statistical significance (*p* = 0.2227). Although the prevalence rates of diabetes mellitus (*p* = 0.143), hypertension (*p* = 0.760), and smoking (*p* = 0.127) in the TTR < 60% group were even higher, it was not statistically significant. In multivariate analysis, female gender (*p* = 0.023) and atrial fibrillation (*p* = 0.011) were associated with TTR < 60%. Multivariate analysis of predictors of TTR < 60% is shown in Table 4.

**TABLE 3:** Univariate analyses of preoperative variables associated with TTR < 60%.

Characteristics	TTR < 60% (*n* = 121)	TTR ≥ 60% (*n* = 153)	*p*
Age, years)	53.3 ± 11.2	51.9 ± 13.8	0.227
Female, n (%)	91(75.3%)	89(58.2%)	0.010
Diabetes mellitus, n (%)	18(14.9%)	14(9.1%)	0.143
Hypertension, n (%)	19(15.7%)	22(14.4%)	0.760
Smoking, n (%)	25(20.7%)	21(13.7%)	0.127
Atrial fibrillation, n (%)	88(68.6%)	82(53.6%)	<0.001
NYHA ≥ III, n (%)	103(85.1%)	102(67.5%)	<0.001
Creatinine, μmoI/L	75.5 ± 16.6	71.3 ± 19.1	0.331
Total cholesterol, mmol/L	3.2 ± 0.8	3.3 ± 0.6	0.757
HbA1c, %	6.3 ± 1.1	5.9 ± 0.9	0.291
Echocardiography			
LVEDD, mm	47.8 ± 4.5	48.2 ± 4.8	0.679
LVEF, %	56.8 ± 6.1	60.5. ± 5.7	0.032
Operations			0.170
MVR, n (%)	66(54.5%)	71(46.4%)	
MVR and AVR, n (%)	34(28.1%)	60(39.2%)	
AVR, n (%)	21(17.4%)	22(14.4%)	
Number of INR values, n	4.6 ± 1.7	5.0 ± 1.5	0.153
weekly doses of warfarin, mg	19.8 ± 6.7	21.2 ± 5.8	0.258

AVR, aortic valve replacement; INR, international normalized ratio; MVR, mitral valve replacement; LVEDD, left ventricular end-diastolic dimension; LVEF, left ventricular ejection fraction; NYHA, New York heart association; TTR, time in the therapeutic range.

**TABLE 4:** Multivariate analysis of predictors of TTR < 60%.

Factors	Odds ratio	95% CI	*p*
Female	1.21	1.08–2.17	0.023
Atrial fibrillation	2.27	1.23–4.29	0.011

In this study, the average duration was 16.7 ± 28.1 months. There were three major bleeding events during follow-up: two cases of gastrointestinal bleeding and one case of cerebral hemorrhage. The occurrence rate of major bleeding events was 1.6% patient-years. There were two thromboembolic events: one case of cerebral embolism and one case of atrial thrombosis. The occurrence rate of thromboembolic events was 1.1% patient-years. There was one case of death which resulted from cerebral hemorrhage. The incidence of death was 0.5% patient-years. The difference in anticoagulation-related complications between the TTR < 60% group and the TTR ≥ 60% group was not statistically significant (*p* = 0.090, Table 5).

**TABLE 5:** Characteristics of warfarin treatment during the study.

Characteristics	All patients (*n* = 274)	TTR < 60% (*n* = 121)	TTR ≥ 60% (*n* = 153)	*p*
Number of INR values	4.8 ± 1.6	4.6 ± 1.7	5.0 ± 1.5	0.153
Weekly doses of warfarin	20.6 ± 6.0	19.8 ± 6.7	21.2 ± 5.8	0.258
Mean monitoring interval, days	53.6 ± 27.1	58.1 ± 33.7	51.8 ± 20.1	0.069
Anticoagulation follow-up times, months	16.7 ± 28.1	18.9 ± 31.4	15.3 ± 26.8	0.171
Anticoagulation-related complications, n (%)	6(2.2%)	4(3.3%)	2(1.3%)	0.687
Major bleeding events, n	3	2	1	
Thromboembolic events, n	2	1	1	
Death, n	1	1	0	

INR, international normalized ratio; TTR, time in the therapeutic range.

## Discussion

In China, keeping high frequency (4-week intervals) of INR monitoring after discharging from the hospital is a significant challenge due to geographical restriction, limited economic resource, and less medical knowledge (18). To reduce patients’ burdens of anticoagulant therapy, a longer interval of 12 weeks was used for patients with stable warfarin doses (5, 7). Studies have shown that longer intervals (> 4 weeks) for INR monitoring are non-inferior to every 4 weeks in TTR with the incidence of anticoagulant complications (7). Meanwhile, owing to the dietary habit and pharmaceutical interferon, the short interval of 4 weeks will more than likely result in more unnecessary dose adjustment, which further destabilized the anticoagulant level (18, 19).

In our study, the average TTR for patients with a 12-week INR follow-up interval has reached to 63.7%. A majority of the previous studies have reported that it ranges between 40 and 78% (11, 12, 20, 21), even under clinical trials with point-of-care home monitoring or computer assisting dose system (11, 22). According to the previous studies, the anticoagulation-related complication is associated with TTR. Yet, there is no specific benchmark of TTR being regarded as completely safe (23, 24). In a study, a TTR of greater than 65% indicated an effective anticoagulation strategy. There existed a target threshold TTR (estimated between 58 and 65%) below which anticoagulation had little benefit (25). Another study found a reduction in the risk of stroke in patients with TTR over 60% and an improved survival rate in patients with TTR over 40% (24). According to Lee and colleagues (14), 90 patients who receive warfarin therapy with atrial fibrillation had an average TTR of 60.6%. In a study published by Hong and colleagues (26), 1,230 AF patients aged 70.1 ± 9.7 years were involved, and their mean TTR was only 49.1%. To sum up, compared with these studies, TTR in our study was acceptable. However, this result may be the consequence of sampling difference. In this study, a stable warfarin dose was administered to all participants for more than 12 months. Patients of not monitoring regularly, taking amiodarone, ethnic minorities, and younger than 18 years old were excluded as they may have a significant effect in anticoagulation quality.

This study has demonstrated that female gender and atrial fibrillation are associated with TTR < 60%. Avarello and colleagues (27) found a lower TTR in females than males in a large population of anticoagulated patients followed at a University hospital anticoagulant clinic. They indicated female is an independent predictor of poor TTR. Henderson and colleagues (28) reported female gender was associated with a 10.1% decrease in TTR. Apostolakis et al. (29) evaluated factors affecting quality of anticoagulation control among patients with vitamin K antagonist. They indicated that female gender were also more likely to have poor TTR values.

Although the occurrence rate of major bleeding and thromboembolic events was lower (1, 3), we do not think the management of anticoagulants in our study is better than others. Some studies showed that anticoagulation-related complications occurred more frequently in the first 6 months after surgery, especially bleeding events (30, 31). Besides that, it may be associated with the different criteria of bleeding and thromboembolic events or patient’s medical knowledge (3, 30). Therefore, a low incidence rate of anticoagulation-related complications was found in our study, and it is not enough to evaluate the quality of patients.

There are some limitations in this study. First, it is a retrospective research and is a single-center study. Second, the result of TTR in this study is sampled from not a large number of patients. Third, the result of TTR is based on patients who had at least two INR values, and not all patients were included in the whole observation period. This point may also influence the results of TTR (20).

## Conclusion

The study used TTR to evaluate the quality of anticoagulation management. There reached an acceptable result of TTR for patients with 12-week INR follow-up interval in stable PT monitoring results. We also found that female gender and atrial fibrillation were associated with TTR < 60%. However, future well-designed prospective studies with a large sample size and detailed analyses of anticoagulation-related complications are still warranted to confirm our findings.

## Data availability statement

The original contributions presented in this study are included in the article/supplementary material, further inquiries can be directed to the corresponding author.

## Ethics statement

The studies involving human participants were reviewed and approved by Henan Province People’s Hospital. The patients/participants provided their written informed consent to participate in this study.

## Author contributions

XZ and XX conceived and designed the experiments. XZ and SW performed the experiments. XZ and XC analyzed the data. XZ, GL, and XL contributed reagents, materials, and analysis tools. XZ wrote the manuscript. All authors contributed to the article and approved the submitted version.
